# Phytoplasma infection in tomato is associated with re-organization of plasma membrane, ER stacks, and actin filaments in sieve elements

**DOI:** 10.3389/fpls.2015.00650

**Published:** 2015-08-19

**Authors:** Stefanie V. Buxa, Francesca Degola, Rachele Polizzotto, Federica De Marco, Alberto Loschi, Karl-Heinz Kogel, Luigi Sanità di Toppi, Aart J. E. van Bel, Rita Musetti

**Affiliations:** ^1^Department of Phytopathology and Applied Zoology, Justus Liebig UniversityGiessen, Germany; ^2^Department of Life Sciences, University of ParmaParma, Italy; ^3^Department of Agricultural and Environmental Sciences, University of UdineUdine, Italy

**Keywords:** actin, BiP protein, endoplasmic reticulum, phloem, phytoplasmas, plasma membrane, sieve elements

## Abstract

Phytoplasmas, biotrophic wall-less prokaryotes, only reside in sieve elements of their host plants. The essentials of the intimate interaction between phytoplasmas and their hosts are poorly understood, which calls for research on potential ultrastructural modifications. We investigated modifications of the sieve-element ultrastructure induced in tomato plants by ‘*Candidatus* Phytoplasma solani,’ the pathogen associated with the stolbur disease. Phytoplasma infection induces a drastic re-organization of sieve-element substructures including changes in plasma membrane surface and distortion of the sieve-element reticulum. Observations of healthy and stolbur-diseased plants provided evidence for the emergence of structural links between sieve-element plasma membrane and phytoplasmas. One-sided actin aggregates on the phytoplasma surface also inferred a connection between phytoplasma and sieve-element cytoskeleton. Actin filaments displaced from the sieve-element mictoplasm to the surface of the phytoplasmas in infected sieve elements. Western blot analysis revealed a decrease of actin and an increase of ER-resident chaperone luminal binding protein (BiP) in midribs of phytoplasma-infected plants. Collectively, the studies provided novel insights into ultrastructural responses of host sieve elements to phloem-restricted prokaryotes.

## Introduction

Phytoplasmas are biotrophic plant-pathogenic wall-less prokaryotes (class *Mollicutes*), phylogenetically related to the low G + C Gram-positive bacteria (Weisberg et al., 1989). Phytoplasmas are associated with several 100s of diseases affecting important crops including ornamentals, vegetables, and fruit trees (Lee et al., 2000). They occur restricted to the sieve elements of host plants and are transmitted to other plants *via* sieve-tube sap feeding leafhoppers (*Cicadellidae*), planthoppers (*Cixiidae*) or psyllids (*Psyllidae*) in a persistent manner (Hogenhout et al., 2008).

Plant–phytoplasma interactions have been poorly characterized due to a lack of techniques. Thus far, it has been impossible to transform or genetically modify phytoplasmas, or simply isolate different strains from mixtures present in nature (Seemüller et al., 2013). Methods for *in vitro* culture of phytoplasmas await further confirmation of feasibility (Contaldo et al., 2012).

Infection of plants by phytoplasmas leads to massive changes in phloem physiology associated with a severely impaired assimilate translocation (Musetti et al., 2013). This leads to characteristic symptoms such as low productivity, stunting, general decline, and reduced vigor of the host (Kartte and Seemüller, 1991). While the macroscopic consequences of phytoplasma activity on host plants have been amply described (Bertaccini, 2007), the effects phytoplasma infection on the ultrastructure of the host cells have been insufficiently examined. In particular, crucial phytopathogenic traits such as adhesion ability to sieve-element membrane (as assumed by Seemüller et al., 2013), as well as the relationship with the sieve endoplasmic reticulum (SER) and sieve-element actin have not yet been studied. Since phytoplasmas probably may exert their action on plants by binding to sieve-element components (Christensen et al., 2005), this study focused on the ability of phytoplasmas to interact with the sieve-element plasma membrane, SER, and sieve-element actin. Resin-embedded leaf sections of healthy and stolbur-affected tomato (*Solanum lycopersicum*) plants [the disease associated with the ‘*Candidatus* Phytoplasma solani’ (‘*Ca*. P. solani’)] were examined by transmission electron microscopy (TEM) combined with immunogold labeling (Musetti et al., 2002).

By western blot analyses of protein extracts from midribs of healthy and ‘*Ca*. P. solani’-infected plants, expression levels of actin and ER-resident chaperone BiP (luminal binding protein) – the latter was chosen as marker of the ER-stress response (Lee, 2005) – were quantified.

The studies provided evidence that infection of *S. lycopersicum* with ‘*Ca.* P. solani’ leads to abnormalities in the sieve-element plasma membrane – SER – actin network. Intimate structural links between phytoplasma body and host cell membranes seem to point to a complex interplay between host and invader during phytoplasma infection.

## Materials and Methods

The preparation of plant material and the microscopy analyses have been performed according the methods previously reported by Buxa (2014).

### Plant Material and Phytoplasma Inoculation

Four *S. lycopersicum* plants (‘cv Micro-Tom’) were infected with the stolbur phytoplasma ‘*Ca.* P. solani’ (subgroup 16 SrXII-A, Quaglino et al., 2013) by grafting. Shoot tips from naturally infected tomato plants, grown in the field, were used as scions and grafted onto 50-days-old healthy tomato plants, in a greenhouse (27°C day, 20°C night). Four 50-days-old, uninfected tomato plants, grown in a greenhouse, were also grafted using shoot tips from healthy plants, and served as controls. Analyses were performed with the advent of symptoms, when plants were three and half months old.

Phytoplasma presence was assessed in randomly collected leaf samples by real time RT-PCR analyses. Total RNA was extracted from 1 g of frozen leaf midribs using RNeasy Plant Mini Kit (Qiagen GmbH, Hilden, Germany). RNAs were reverse-transcribed using a QuantiTect Reverse Transcription Kit (Qiagen GmbH, Hilden, Germany) with random hexamers, following the manufacturer’s instructions. Real time RT-PCR analyses were performed using the primers 16S stol F2/R3 based on the 16SrRNA gene of ‘*Ca*. P. solani’ (accession n° AF248959, Santi et al., 2013a). Real time RT-PCR reactions were set up with 2X Sso Fast^TM^ Eva Green^®^ Supermix (Bio-Rad Laboratories Co., Hercules, CA, USA), primers at 400 nM each, and 10 ng of cDNA in a total volume of 10 μl. The reactions were performed in a CFX96 Real Time PCR Detection System (Bio-Rad Laboratories Co., Hercules, CA, USA) using the following conditions: 95°C for 2 min, 40 cycles of 95°C for 15 s and 60°C for 1 min. The melting curve was performed with a ramp from 60 to 95°C.

### Conventional Transmission Electron Microscopy

Fifteen randomly chosen leaf midrib segments, sampled from the four either infected or healthy tomato plants, were cut into pieces 6–7 mm in length, fixed in a solution of 3 % glutaraldehyde in 0.1M phosphate buffer (PB), pH 7.2, for 2 h at 4°C, washed for 30 min at 4°C in PB and post-fixed for 2 h with 1% (w/v) OsO_4_ in PB at 4°C (Musetti et al., 2011). Samples were dehydrated in ethanol and propylene oxide, embedded in Epon/Araldite epoxy resin (Electron Microscopy Sciences, Fort Washington, PA, USA). Serial ultrathin sections (60–70 nm) of about 60 samples from each healthy or infected plant, were cut using an ultramicrotome (Reichert Leica Ultracut E ultramicrotome, Leica Microsystems, Wetzlar, Germany) and collected on 200 mesh uncoated copper grids, stained and then observed under a Philips CM 10 (FEI, Eindhoven, The Netherlands) TEM operating at 100 kV.

### Sample Preparation for Electron Microscopy of Immuno-Labeled Sections

Fifteen randomly chosen leaf midrib segments were excised from infected or healthy tomato plants. Segments were cut into small portions (6–7 mm in length), fixed in 0.2% glutaraldehyde, rinsed in 0.1 M PB, pH 7.4 and dehydrated in graded ethanol series (25-, 50-, 75%, 30 min for each step) at 4°C. After 1 h of the final 100% ethanol step, the samples were infiltrated in a hard-grade London Resin White (LRW; Electron Microscopy Sciences, Fort Washington, PA, USA)-100% ethanol mixture in the proportion 1:2 for 30 min, followed by LRW:ethanol 2:1 for 30 min, and 100% LRW overnight at room temperature (with a change 1 h after the start of the infiltration). The samples were embedded in Eppendorf tubes using fresh LRW containing benzoyl peroxide 2% (w/w) according to manufacturer’s protocol and polymerized for 24 h at 60°C (Musetti et al., 2002).

### Immunogold Labeling

Several serial ultrathin sections (60–70 nm) of about 60 LR-White-embedded samples from each healthy or infected plant were cut using an ultramicrotome (Reichert Leica Ultracut E ultramicrotome, Leica Microsystems, Wetzlar, Germany) and collected on carbon/formvar coated 400 mesh nickel grids (Electron Microscopy Sciences, Fort Washington, PA, USA). To visualize the presence and distribution of actin in LR-White-embedded plant tissue, immunogold-labeling technique was performed (modified after White et al., 1994). Unspecific binding sites, were blocked placing grids carrying the sections on droplets of blocking solution containing normal goat serum (NGS) diluted 1:30 in 1% BSA in PBS, pH 7.6, for 2 h at room temperature. Subsequently, the grids were incubated overnight at 4°C with primary mouse monoclonal antibody against actin (MAB anti-actin, clone C11, Agrisera, Vännäs, Sweden) diluted 1:200 in blocking solution. Control grids were incubated in 1% BSA/PBS without primary antibody. All grids were then rinsed with PBS, and treated for 1 h at room temperature with secondary goat anti-mouse antibody coated with colloidal 5 nm gold particles (GAM 5; Auro Probe EM GAM G5 Amersham, Arlington Heights, IL, USA), diluted 1:40 in 1% BSA/PBS. After staining with 3% uranyl acetate and 0.1% lead citrate (Reynolds, 1963) samples were observed under TEM, as reported above.

To assess the subcellular distribution of actin labeling, immunogold particle number was determined in healthy and infected sieve elements. Gold spots were manually counted and recorded on plasma membrane, cytoplasm (i.e., mictoplasm, Hafke et al., 2013) and lumen of three sieve elements in three not-serial sections (Bamunusinghe et al., 2009).

### Western Blot Analyses

Total proteins were extracted from *S. lycopersicum* midribs: 150 mg of fresh tissue from three healthy and three stolbur-diseased plants were frozen in liquid nitrogen with 200 ml of glass microbeads (diameter 200 mm), ground to a powder with a dental amalgamator (TAC 200/S Amalgamator, Linea TAC, Italy), and resuspended in 300 μl of lysis buffer [50 mM TRIS-HCl pH 7.5, 2 M thiourea, 7 M urea, 2% (v/v) Triton X-100, 1% dithiothreitol (DTT), 2% (w/v) polyvinylpolypyrrolidone (PVP), 1 mM PMSF, 0.2% β-mercaptoethanol]. Samples were centrifuged at 15000 × *g* for 20 min at 4°C, then the supernatant was recovered and subjected to a second centrifugation (15000 × *g* for 20 min at 4°C). The protein concentration was assessed according to Bradford (1976) using bovine serum albumin as standard (BSA, Sigma, USA). For each sample 20 μg of total protein was separated in a 12% acrylamide SDS-PAGE (Laemmli, 1970) and blotted at 100 V for 60 min to a nitrocellulose membrane (GE Healthcare Bio-Sciences AB, Uppsala, Sweden) using a Mini Trans-Blot cell apparatus (Bio-Rad Laboratories, Hercules, CA, USA). Both protein loading and transfer efficiency were verified by Ponceau-S staining. For quantitative analysis of actin and ER stress-sensor BiP, western blot analyses were performed with polyclonal antibodies raised against *Arabidopsis thaliana* actin (AS132640; Agrisera AB, Vännäs, Sweden) and BiP luminal-binding protein (AS09481; Agrisera AB, Vännäs, Sweden) diluted 1:2500 and 1:10000, respectively. Membranes were blocked 1 h in PBS 5% (w/v) skim milk, probed with primary antibodies for 1 h and with anti-rabbit IgG horseradish peroxidase conjugated antibody (GE Healthcare Bio-Sciences AB, Uppsala, Sweden) for 1 h. Chemiluminescence detection was assessed with Pierce ECL Plus Western blot Substrate system (Pierce Biotechnology, Rockford, IL, USA), according to manufacturer’s instructions.

### Quantification and Statistical Analysis

An ANOVA procedure with SPSS software version 21.0 (IBM SPSS Statistics, Armonk, NY, USA) evaluated differences in the number of gold particles observed in healthy and infected sieve elements. Homogeneity of variance and distributional assumptions were assessed via the Levene test. A significance level of 0.05 was used for all comparisons.

A densitometric analysis was conducted on actin and BiP Western Blot signals with Quantity One 4.6.6. Bio-Rad Software (Bio-Rad Laboratories, Hercules, CA, USA). A total of six samples for each plant were analyzed. The statistical analysis of densitometric values was performed with the unpaired *t*-test.

## Results

### Plant Symptom Development and Phytoplasma Molecular Detection

**FIGURE 1:**
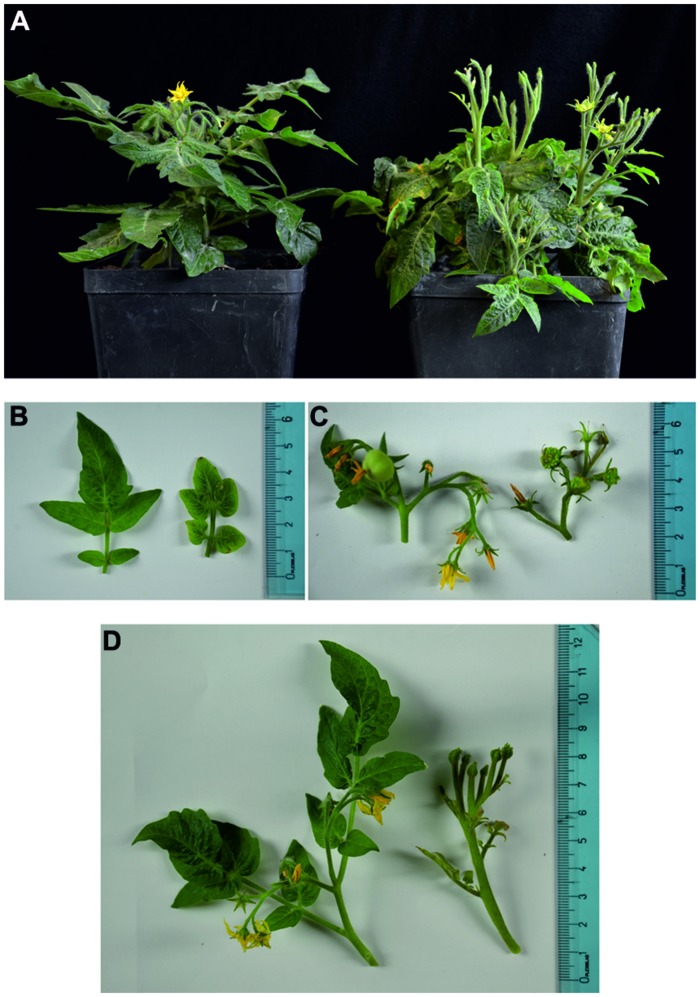
**(A–D)** Images of healthy (left half of the panels) and stolbur-infected (right half) tomato Micro-Tom plants. Healthy tomato plants show regular growth, normal leaves and flowers are present, whereas in stolbur-infected plants diffuse symptoms are visible **(A)**. Leaf blades are severely reduced **(B)**. Buds are aborted; flowers are malformed with green petals **(C)**. Shoots develop witches’ brooms and show a stunted habit **(D)**.

Control plants were regularly grown, without disease symptoms. In stolbur-infected plants, typical symptoms, such as leaf yellowing, leaf-size reduction, witches’ brooms and stunting, emerged nearly 2 months after grafting (**Figure 1**). Real time RT-PCR of ‘*Ca*. P. solani’ *16SrRNA* confirmed the presence of phytoplasmas in leaf samples from stolbur-infected *S. lycopersicum* before treatment for microscopic examination. Starting from 40 ng of total cDNA, stolbur phytoplasma *16SrRNA* was amplified in infected plants, whereas no amplification of the *16SrRNA* gene was obtained in control plants (**Table 1**).

**Table 1:** Molecular detection of ‘*Candidatus* Phytoplasma solani’ in tomato plants.

Well	Label	Primers	C_q_
F1	Stolbur-infected *S. lyc* 1	16SRT f2r3	17.98
F2	Stolbur-infected *S. lyc* 2	16SRT f2r3	19.38
F3	Stolbur-infected *S. lyc* 3	16SRT f2r3	17.54
F4	Stolbur-infected *S. lyc* 4	16SRT f2r3	16.10
F5	C. roseus Stol+	16SRT f2r3	17.55
F6	Grapevine Stol+	16SRT f2r3	23.50
F7	Tomato C–	16SRT f2r3	None
F8	H_2_O	16SRT f2r3	None

*Stolbur phytoplasma *16SrRNA* was detected in infected plants, whereas no amplification was obtained in control plants.*

*Cq: quantification cycle (the cycle in which the threshold line intersects the amplification curve of the sample in the exponential phase of the reaction).*

*F1–F4: stolbur-infected *Solanum lycopersicum* samples.*

*F5: stolbur-infected *Catharanthus roseus*, positive control.*

*F6: stolbur-infected grapevine, positive control.*

*F7: healthy *S. lycopersicum*, negative control.*

### Sieve-Element Membrane Structures in Control and Infected Plants

**FIGURE 2:**
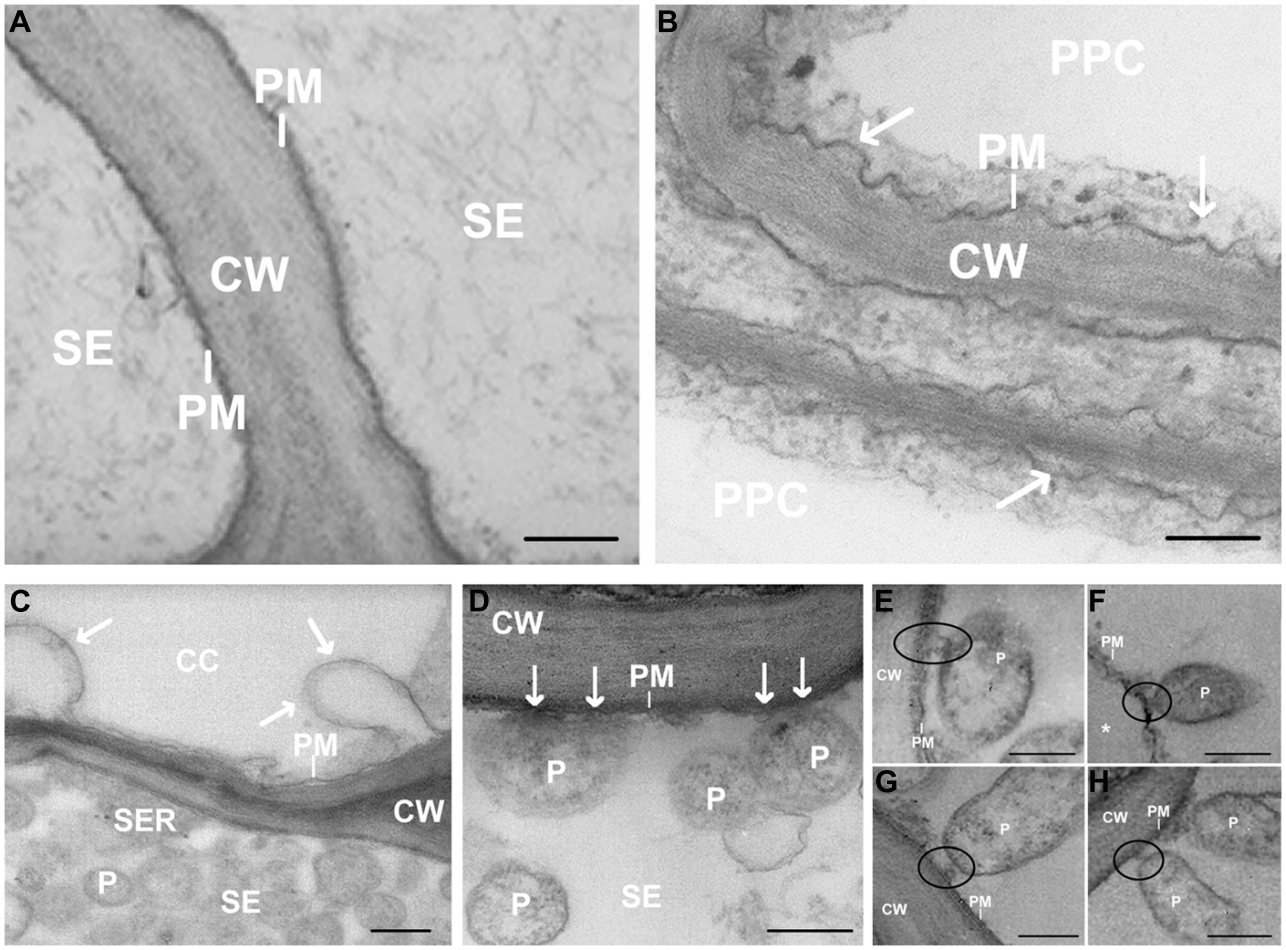
**(A–H)** Transmission Electron Microscopy (TEM) micrographs of main vein cross-sections of healthy **(A)** and stolbur-diseased tomato leaves **(B–H)**. Arrows in **(B–D)** indicate cell membrane disorganization and black circles in **(E–H)** show attachment of phytoplasma body to sieve-element plasma membrane. ^∗^ indicates a detachment of the SE plasma membrane from the wall. CW, cell wall; P, phytoplasma; PM, plasma membrane; CC, companion cell; PPC, phloem parenchyma cell; SE, sieve element. Scale bars **(A)** = 400 nm; **(B–H)** = 200 nm.

In total, 60 sections from the 15 embedded blocks have been screened by TEM. TEM images revealed the sieve-element plasma membrane appressed to the cell wall in healthy leaves (**Figure 2A**). In infected samples, the plasma membrane of the phloem cells (phloem parenchyma cells, companion cells and sieve elements – SEs) was deformed, invaginated or undulating (**Figures 2B–H**). The membrane of parietal phytoplasmas and the sieve-element plasma membrane appeared in close contact (**Figure 2D**) *via* a membrane-bound structure forming a firm connection (**Figures 2E–H**). The typical pleomorphism and the ribosomes inside the bacterial bodies (**Figure 2D**) enabled a ready discrimination between phytoplasmas and sieve-element plastids (SEPs) even though size and location were similar (see **Figure 5B** and Ehlers et al., 2000). The characteristic multiple anchoring of SEPs to the sieve-element plasma membrane (Ehlers et al., 2000) was never observed for phytoplasmas.

### Sieve-Element Actin and Sieve Endoplasmic Reticulum and their Connections with Phytoplasma Cells by Transmission Electron Microscopy

**FIGURE 3:**
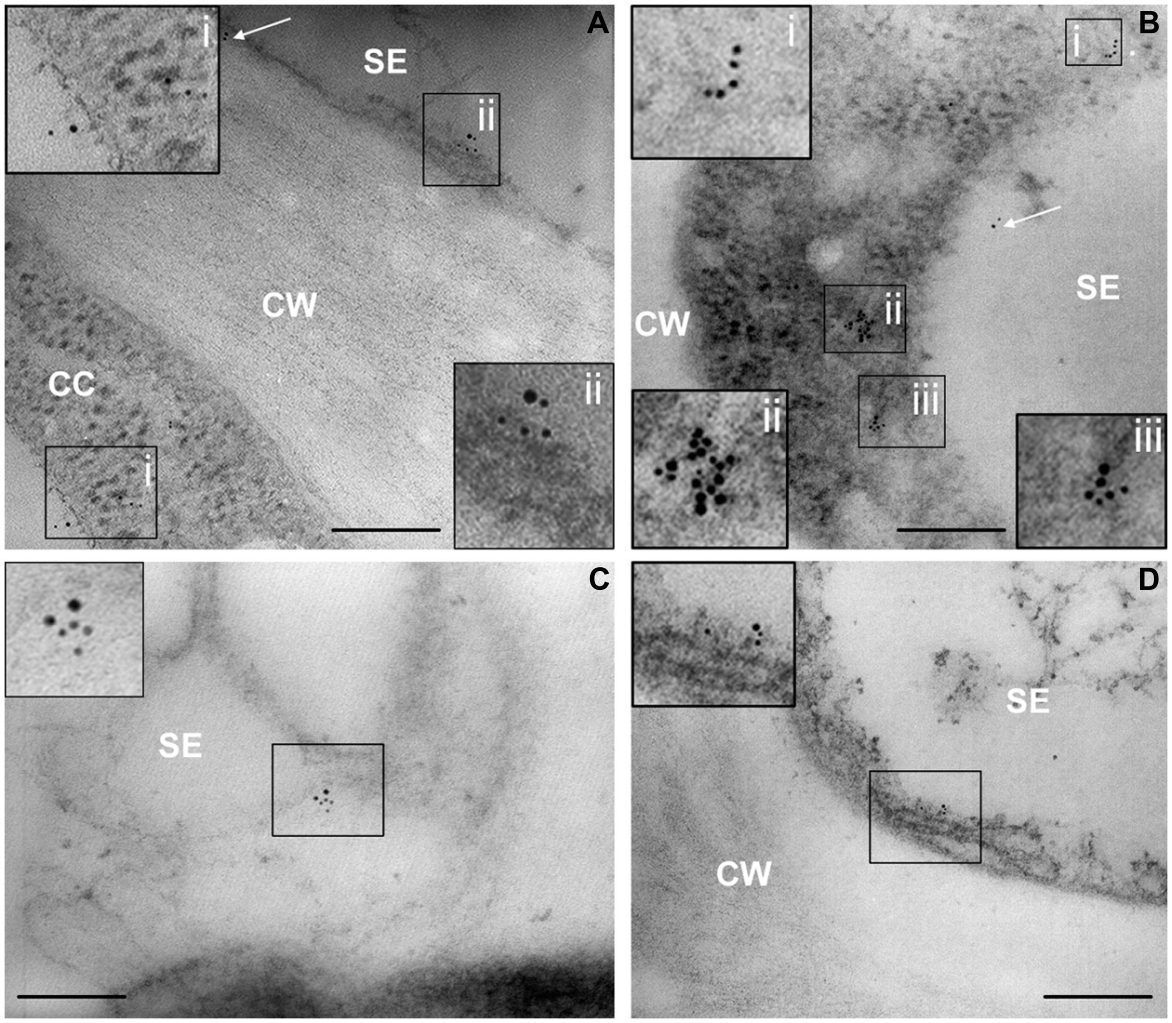
**(A–D)** TEM micrographs of main-vein cross-sections of healthy tomato leaves. Gold labeling of SE actin was clustered at the plasma membrane (**A,D**, squares) and the near cytoplasm area (mictoplasm) of sieve elements (**B,C**, squares). Labeling also occurs on the proximity of cell walls (**A**, arrow), in the lumen of the SEs (**B**, arrow) and in the cytoplasm of the adjacent companion cells **(A)**. In insets, areas of interest of **(A–D)**, are magnified. CC, companion cell; CW, cell wall; SE, sieve element; Scale bars = 200 nm.

Control sections (from both healthy and infected samples), incubated with buffer alone, did not show labeling (not shown). In agreement with labeling with α-actin-gold-conjugated antibodies, actin occurred along the sieve-element membrane (**Figure 3A**), in the sieve-element mictoplasm and lumen (**Figures 3B–D**), and also in companion cell cytoplasm. The existence of an actin network in sieve elements has recently been demonstrated by Hafke et al. (2013).

**FIGURE 4:**
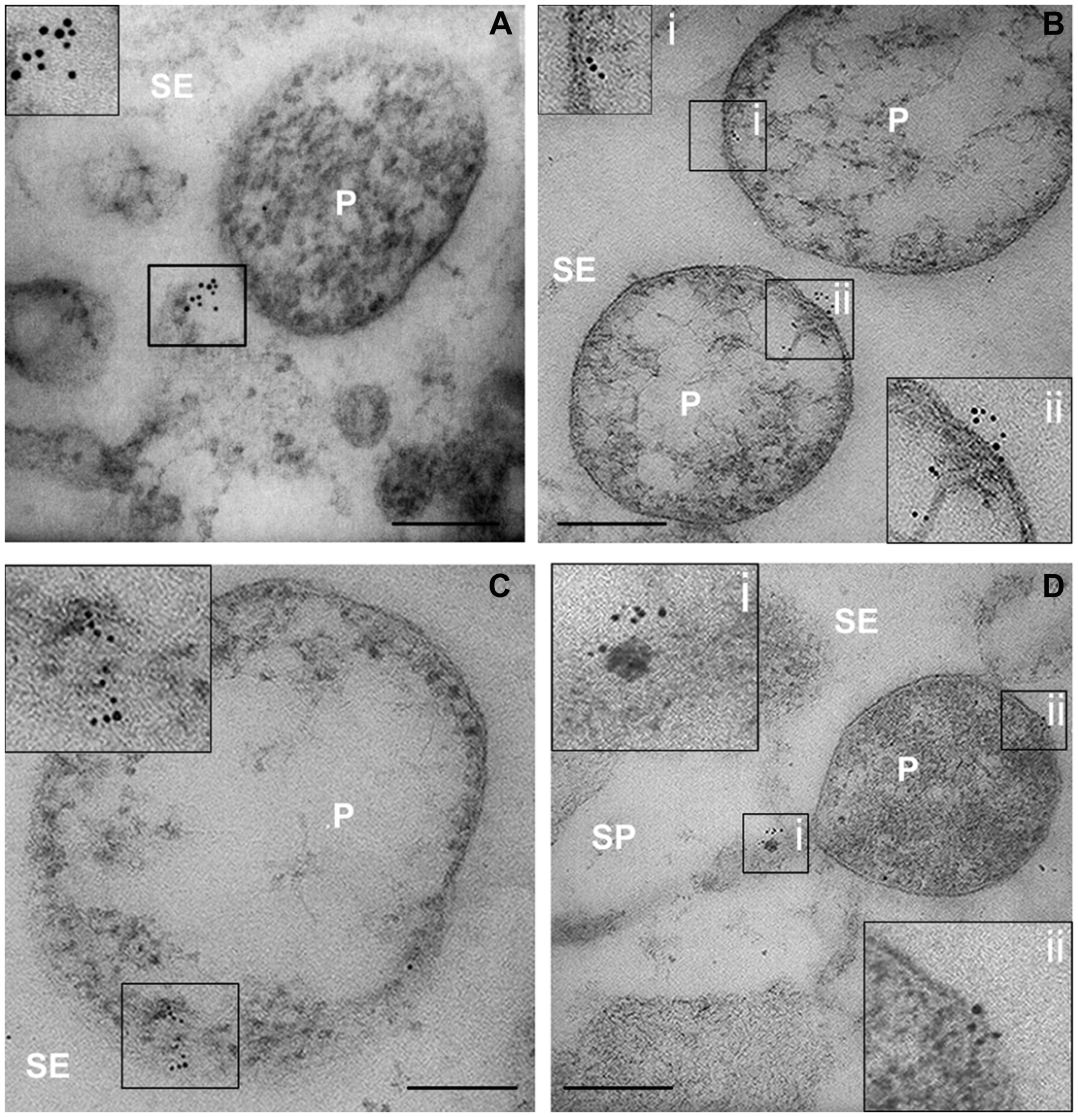
**(A–D)** TEM micrographs of main-vein cross-sections of stolbur-diseased tomato leaves. Aggregated of SE actin in contact with the phytoplasma cells were evidenced by a TEM-immunogold technique. In insets, areas of interest of **(A–D)**, are magnified. P, phytoplasma; SE, sieve element; SP, sieve pore. Scale bars = 200 nm

In infected samples, high spatial resolution images revealed a co-localization of sieve-element actin and phytoplasma cells (**Figures 4A–D**). Ultrastructural images obtained from infected samples indicated that antibody dots exclusively resided in the sieve-element lumen in association with phytoplasma cells and were always aggregated at one side of the phytoplasma membrane surface (**Figures 4A–D**). Within sieve pores too, actin was localized to phytoplasma cells (**Figure 4D**). These actin fields often co-localized with the tubular corridors between phytoplasma body and plasma membrane (e.g., **Figure 4D**).

Gold particles were counted to determine the labeling distribution in sieve-element membrane, mictoplasm and lumen. The countings were statistically analyzed (**Table 2**). In healthy sieve elements, gold particles were mainly found in the mictoplasm and in association with the membrane, whereas in the cell lumen they were significantly less abundant. In infected sieve elements, gold particles were observed, almost exclusively, in the lumen, in association with phytoplasmas. Gold spots in the lumen of phytoplasma-infected sieve elements were significantly more abundant compared to those recorded in the lumen of healthy ones (**Table 2**) which indicates a displacement of actin away from the plasma membrane. It was noteworthy that the absolute number of actin dots per sieve-element cross-section was approximately 35% lower than in control sieve elements (**Table 2**).

**Table 2:** Sieve elements of healthy and infected tomato were analyzed by immunogold labeling and electron microscopy, to assess actin subcellular distribution.

Sample	# Fields	Membrane	Lumen	Mictoplasm	Total gold particles
Healthy	9	12.00 ± 2.35 a	4.78 ± 5.09 b	16.89 ± 6.17 a	303
Infected	9	0.00 ± 0.00	23.11 ± 1.62 c	0.00 ± 0.00	210

*Fields are defined as the cross sections of three sieve elements observed in three non-serial sections. Gold particles in each field were counted manually and determined for the sieve-element plasma membrane, mictoplasm and lumen. Zeros indicate subcellular domains without immunogold label. Different letters next to each standard deviation represent significant differences. *P*-values < 0.05.*

**FIGURE 5:**
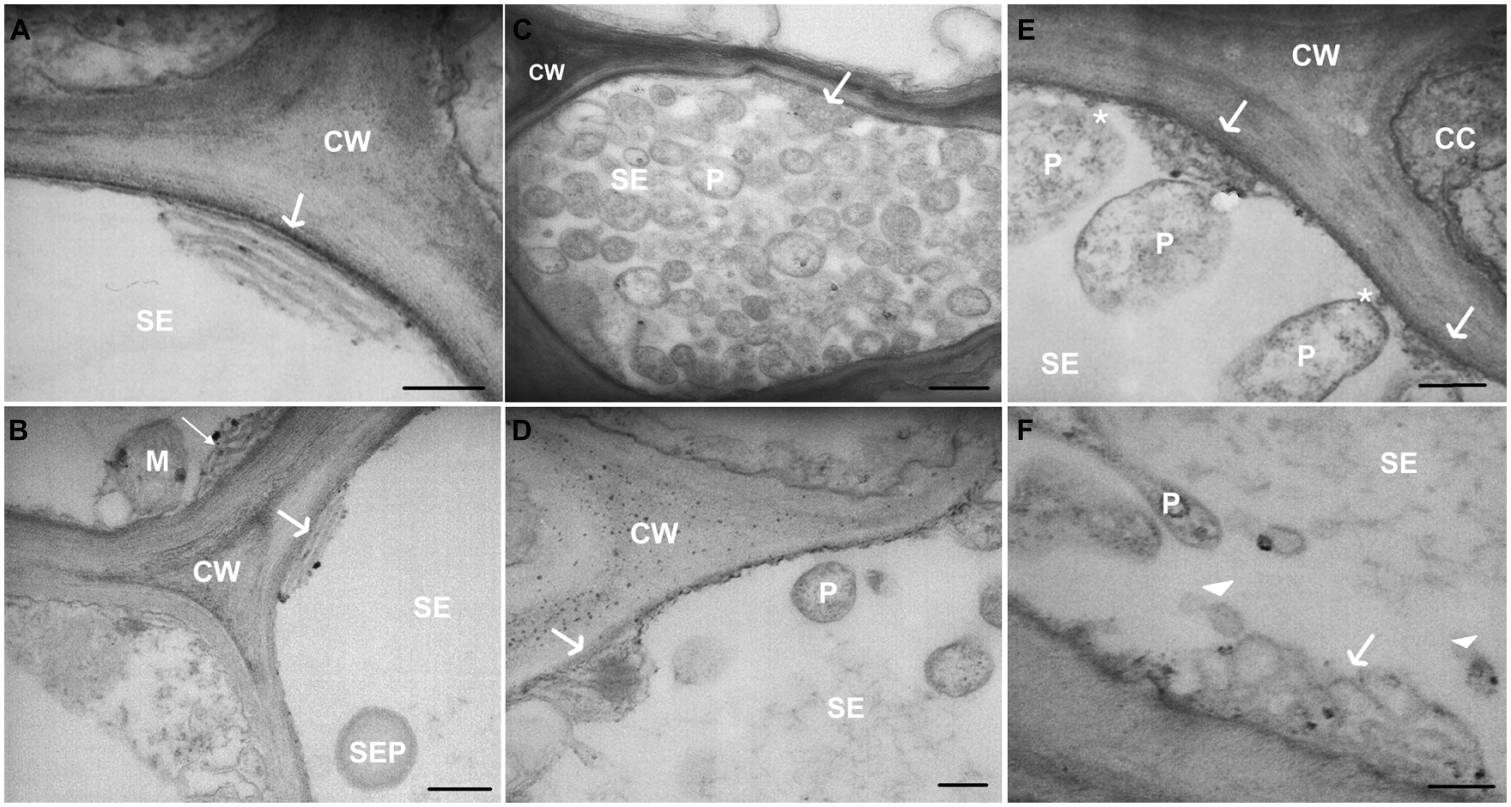
**(A–F)** TEM micrographs of main veins cross-sections of healthy **(A,B)** and stolbur-diseased tomato leaves **(C–F)**. Arrows point to ER organization, asterisks indicate attachment of phytoplasma cell to sieve-element plasma membrane. In stolbur-diseased samples SER cisternae were frequently intruding into the sieve-element lumen **(C–E)** and were fragmented into lobes and vesicles **(F)**. CC, companion cell; CW, cell wall; M, mitochondria; P, phytoplasma; PM, plasma membrane; SE, sieve element; SEP, sieve-element plastid. Scale bars **(A,B,D)** = 200 nm; **(C)** = 400 nm.

TEM images of healthy samples showed SER stacks mostly orientated parallel to the sieve-element plasma membrane (**Figures 5A,B**), while the SER seems to be distorted in stolbur-diseased samples (**Figures 5C–F**). In infected plants, SER stacks frequently were fragmented into lobes and vesicles intruding into the sieve-element lumen (**Figure 5F**). Besides phytoplasmas attached to sieve-element plasma membrane (as above reported and **Figure 5E**) or free-lying in the lumen (**Figure 5C**), phytoplasma cells were located near to the SER (**Figures 5C,E**), but minute anchors attaching to the SER-stacks (as reported for sieve element plastids, Ehlers et al., 2000) were absent. Strikingly, actin labeling was absent on the surface of phytoplasmas adhered to the ER. Two modes of parietal contact seem to occur: adhesion to the ER or tubular contacts with the plasma membrane which probably concur in time (**Figure 5E**).

### Alteration of Actin and BiP Protein Expression in Stolbur-Diseased Tomato Midribs

**FIGURE 6:**
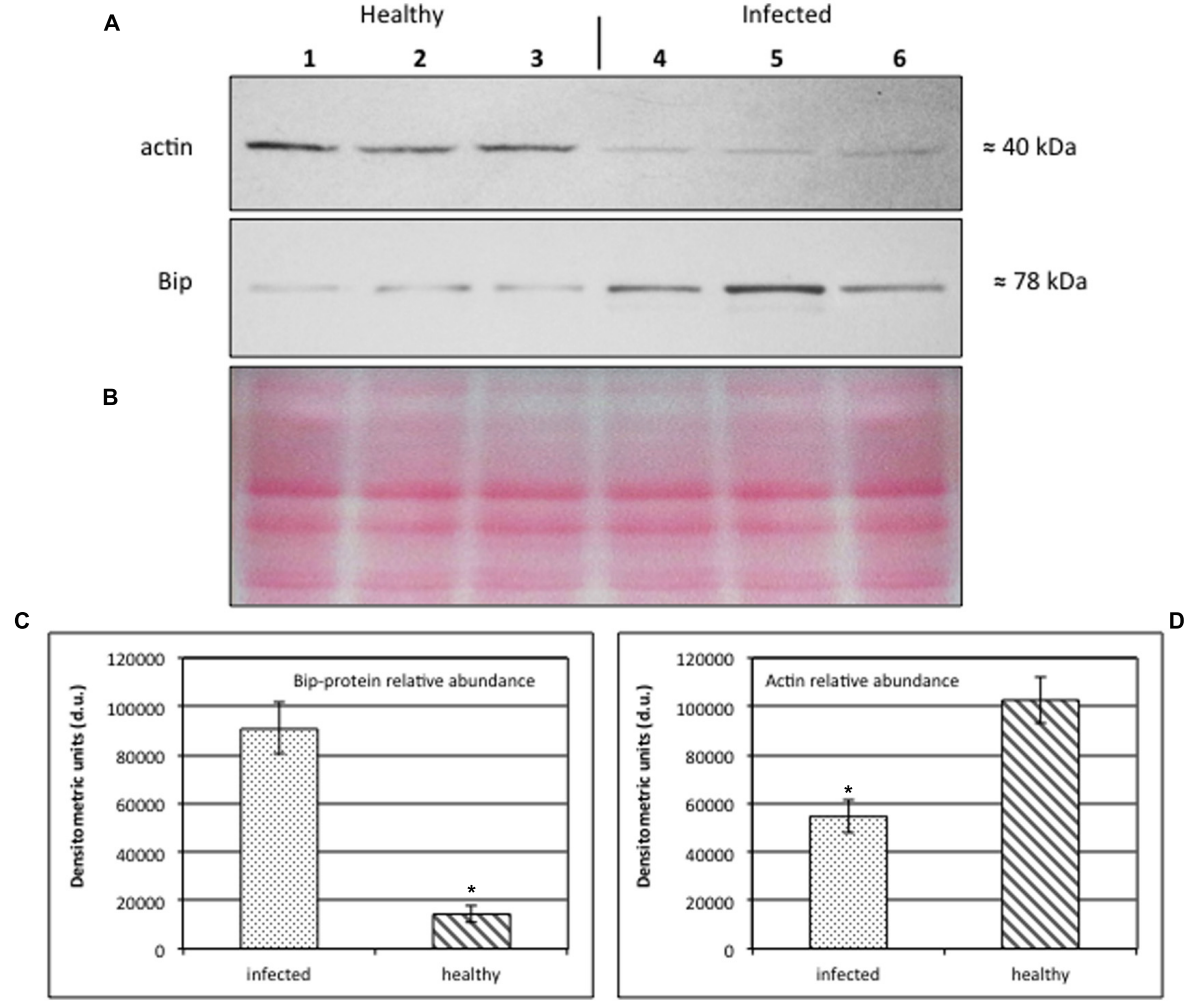
**(A–D)** Western blot analyses of *S. lycopersicum* midrib extracts. Three healthy (lanes 1–3) and three infected (lanes 4–6) plants were analyzed. Immunoreactive signals corresponding to actin and ER luminal binding protein (BiP) displayed the expected molecular weights of ≈40 and ≈78 kDa, respectively **(A)**. Amount of loaded protein were checked by Ponceau-S staining **(B)**. Quantification of immunoreactive luminescence signals was achieved by densitometric scanning of the respective actin **(C)** and BiP **(D)** bands. The mean of six replicates (±SD) was calculated for each sample. Actin *T*-value: –9.977; BiP *T*-value: 15.068. * denotes significant difference of the means (P-values ≤ 0.001).

Western Blot analyses (**Figures 6A–D**) were performed on midrib extracts from healthy and stolbur-diseased plants. The rationale of using midribs is that they contain the sieve elements as the phytoplasma carriers. This approach revealed that actin and BiP protein levels significantly varied in infected plants compared to the healthy ones (**Figure 6A**). Densitometric analyses indicated that the actin level in extracts from infected midribs was significantly lower than in healthy ones (**Figure 6C**). The 40% decrease (**Figure 6C**) is in agreement with the decreased actin contents measured by immuno-gold labeling (**Table 2**). By contrast, infected tissues displayed a 6.3-fold-increased BiP protein level in comparison with healthy samples (**Figure 6D**). The densitometric differences in protein expression of both actin and BiP in healthy and infected plants turned out to be highly significant (*p*-value ≤ 0.001).

## Discussion

It has been advanced that cytological relationships between phytoplasmas and sieve elements are essential for the establishment of pathogenic activity in the host (Christensen et al., 2005). Despite their presumptive importance, structural changes during infection have not been investigated in depth. Past and recent studies hinted at structural modifications of host tissue triggered by phytoplasma infection (Rudzińska-Langwald and Kamińska, 2001; Musetti et al., 2013; Santi et al., 2013b). Moreover different effectors providing communication in phytoplasma–plant and phytoplasma–insect interrelationships, have been described (Hoshi et al., 2007; Bai et al., 2009; Galetto et al., 2011; McLean et al., 2014; Sugio et al., 2014). The present EM studies demonstrate massive structural modifications of infected sieve elements (**Figures 2–5**), which may be accompanied by profound metabolic changes (**Figure 6**).

### Interconnections between Phytoplasmas and Sieve-Element Plasma Membrane

Among the diverse traits of *Mollicutes* infecting humans and animals, adherence to host membranes is regarded as an important pathogenic factor (Razin et al., 1998). Phytoplasmas seem to be in close contact with the sieve-element plasma membrane (Marcone, 2010), but specific adherence structures to host membranes have not been described. Adherence would be feasible, since several studies demonstrated the existence of a subset of adhesin-like membrane proteins in most phytoplasmas (for a review, see Kube et al., 2012; Neriya et al., 2014).

Here, TEM observations evidence major modifications of the plasma membrane in infected sieve elements. Parietally located phytoplasmas do not only adhere to the SER (**Figure 5**), but also form intimate tubular contacts toward the SE plasma membrane (**Figures 2E–H**). In some pictures (**Figures 2H** and **5E**) both forms of contact were observed side by side.

### Interconnections between Phytoplasmas and Host Cytoskeleton

Both animal and plant pathogens actively interact with the host cytoskeleton to successfully enter in the host (Rottner et al., 2005; Pizarro-Cerdá and Cossart, 2006) and move inside host cells (Tilney and Portnoy, 1989; Sansonetti, 1993; Opalski et al., 2005). Phytoplasmas interact with the host cytoskeleton by means of membrane proteins (so-called antigenic membrane proteins, AMP or immunodominant membrane proteins, IMP), capable to bind to the vector (Suzuki et al., 2006; Galetto et al., 2011) or plant actin filaments (Boonrod et al., 2012).

In our study, the connection between the invader phytoplasma and sieve-element actin has been described *in situ*. Apparently, phytoplasmas impose a reorganization that anchors SE actin to the phytoplasma surface. As already known for other prokaryotes (Lybarger and Maddock, 2001; Dworkin, 2009), the connection between actin and phytoplasma turned out to be unilateral, indicating a polarity in the phytoplasma body. Unipolar acquisition and polymerization of host actin has evolved, in particular, in Gram-positive intracellular pathogenic bacteria, such as *Listeria monocytogenes*, to facilitate cell-to-cell spread (Lybarger and Maddock, 2001). A similar asymmetric polymerization of host actin may occur in phytoplasma-infected sieve elements to enable bacterial movement (Rudzińska-Langwald and Kamińska, 1999). Likewise, interaction between phytoplasmas and host actin (Boonrod et al., 2012) may facilitate bacterial spread through the narrow sieve pores *via* pleomorphic modification of phytoplasma *corpus*, as indicated by concurrent gold labeling of sieve-plate areas and phytoplasmas (**Figure 4D**). Phytoplasmas do not appear to possess actin thus far (Christensen et al., 2005), but they have contractile membrane proteins (Kakizawa et al., 2006), which might help to pass the sieve pores, which have smaller diameters (e.g., van Bel, 2003) than those of phytoplasmas (Hogenhout et al., 2008). In plant cells, movement of organelles, including plastids, depends on their interaction with cytoskeleton and ER (Schattat et al., 2011). Phytoplasmas being in the size range of plastids may use a similar actin-based mechanism of displacement.

To gain additional evidence for actin involvement in the sieve-element interaction with phytoplasmas, quantitative actin expression analyses were carried out. Western blotting and gold labeling demonstrated that the interaction between phytoplasmas and actin in infected sieve elements is associated with a decrease of the amount of actin. This interpretation should be made with care, as it departs from the assumption that the changes occur in sieve elements, the exclusive location of phytoplasmas. It is not excluded that part of the changes occurs in the surrounding (vascular) cells given the use of entire midribs. Nevertheless, a similar reduction of actin content in infected cells measured in expression studies (**Figure 6**) and in immuno-labeling studies (**Table 2**) render credibility to the view that the values obtained with midribs hold for sieve elements.

Dynamic actin re-arrangement is regulated by a pool of actin-binding proteins, named actin depolarizing factors (ADFs), which sense stresses and environmental modifications and regulate the cytoskeleton through diverse biochemical activities (Hussey et al., 2006; Staiger and Blanchoin, 2006), responsible for actin turnover. High levels of ADFs confer high severing frequencies and decreased actin filament lengths and lifetimes (Henty et al., 2011) and, hence a dramatic decrease of the overall number of actin filaments (Thomas et al., 2006). These events would explain the decreased western blot signals in infected tissue.

In stolbur-diseased plants, ADF genes have been reported significantly overexpressed (Hren et al., 2009). The decrease of polymerized actin might be correlated with an activation of host defense mechanisms, since actin depolymerization is a potential inductor of plant defense responses (Kobayashi and Kobayashi, 2007). Although the manner in which actin disruption is linked with the defense response is unclear given the dynamic behavior of actin filaments in immune responses, evidence is accumulating that actin participates in cellular signaling cascades in phytoplasma–host interaction (Boonrod et al., 2012).

Furthermore, ADF activates actin-based motility of bacteria, as reported for *Listeria monocytogenes* (Carlier and Pantaloni, 2007) increasing the rate of propulsion by shortening the actin tails. Shorter actin filaments could therefore be of advantage for phytoplasma movement along the sieve-element cytoskeleton.

### Phytoplasma Effects on the Structure of the Sieve-Element Reticulum

Rudzińska-Langwald and Kamińska (2001) observed that SER was often situated in the sieve-element lumen being separated from the plasma membrane and in close association with phytoplasmas in sieve elements of *Limonium sinuatum* infected by Aster Yellows. Here (**Figure 5**), SER undergoes a re-organization in infected sieve elements accompanied by a deformation of the SER stacks, resulting in expansion of the cisternae, development of the lobes and fragmentation into vesicles. Such morphological modifications have been described as part of the “unfolded protein response” (UPR; Bernales et al., 2006), characterized by the accumulation of unfolded proteins in the SER. External stimuli such as pathogen invasion, nutrient deficiency and other environmental factors exert stress on the cellular metabolism leading to aberrations in Ca^2+^ or redox regulation and protein synthesis. These responses enhance the level of misfolded proteins in the ER and trigger UPR (Ye et al., 2011). The UPR is considered important to recover the normal function of ER, to mitigate the stress exerted on the ER, and to prevent the cytotoxic impact of malformed proteins (Jelitto-Van Dooren et al., 1999; Xu et al., 2005; Slepak et al., 2007; Urade, 2007; Preston et al., 2009). In both mammals and plants, the UPR mechanism also includes increased synthesis and activity of several ER-resident proteins, such as the ER chaperone BiP. We found a sevenfold increase of BiP protein levels in stolbur-diseased plants. This suggests a phytoplasma-triggered UPR similar to what has been reported for tobacco infected with Potato Virus X (Ye et al., 2011).

## Conclusion

In conclusion, our results show that stolbur-phytoplasma infection results in a significant re-organization of the sieve-element ultrastructure in phloem tissue of *S. lycopersicum*.

Despite the structural interconnections between phytoplasmas and the host sieve-element plasma membrane and actin and the massive impact of phytoplasma infection on the SER ultrastructure, the functional nature of the interactions remains largely unclear. The changes probably express a transformation that benefits growth, maintenance and transport of phytoplasmas. Phytoplasmas may effectively re-arrange the host ultrastructure to enable nutrient supply and systemic spread *via* the sieve elements, which enables a fast distribution and proliferation of bacteria inside the host plant. On the other hand, the extensive re-organization of the membrane systems and actin network in sieve elements provoked by ‘*Ca*. P. solani’ may also be a protective answer of the plant to ensure fast defense reactions and signaling. Unlike other plant cells, the sieve elements do not contain several significant organelles (e.g., Knoblauch and van Bel, 1998) indispensable for most plant immune responses, so the release of effector proteins by phytoplasmas into other phloem cells (Bai et al., 2009) *via* plasmodesmata might induce a profound alteration of the host sieve elements.

## Author Contributions

RM and SB conceived the project under the supervision of AvB and KK, SB, and RM established the protocols for the electron microscopy and performed microscopical observations. AL prepared infected tomato plants. FDM and RP performed phytoplasma detection by real-time RT-PCR on tomato leaves. FD and LS established the protocols and performed western blot analyses. SB and RM wrote the manuscript with extensive support of AvB.

## Conflict of Interest Statement

The authors declare that the research was conducted in the absence of any commercial or financial relationships that could be construed as a potential conflict of interest.
